# Analysis of the Correlation Between Cardiac Markers in Post-Mortem Vitreous Humor and the Perimortem Agony Interval

**DOI:** 10.3390/ijms26072996

**Published:** 2025-03-25

**Authors:** Matteo Antonio Sacco, Valerio Riccardo Aquila, Saverio Gualtieri, Maria Cristina Verrina, Lucia Tarda, Alessandro Pasquale Tarallo, Angela Carbone, Francesco Ranno, Pietrantonio Ricci, Isabella Aquila

**Affiliations:** Institute of Legal Medicine, Department of Medical and Surgical Sciences, “Magna Graecia” University, 88100 Catanzaro, Italy; matteoantoniosacco@gmail.com (M.A.S.); valerioriccardo.aquila@studenti.unicz.it (V.R.A.); saverio.gualtieri@studenti.unicz.it (S.G.); mariacristina.verrina@studenti.unicz.it (M.C.V.); lucia.tarda@studenti.unicz.it (L.T.); alessandropasquale.tarallo@studenti.unicz.it (A.P.T.); angela.carbone1@studenti.unicz.it (A.C.); francesco.ranno@studenti.unicz.it (F.R.); ricci@unicz.it (P.R.)

**Keywords:** time since deposition, forensic sciences, bloodstains

## Abstract

Forensic biochemistry has often relied on the vitreous humor as a matrix for toxicological investigations due to its stability and isolation from post-mortem redistribution processes. Recently, the scope of research has expanded to explore the vitreous humor as a medium reflecting systemic and pathological changes, particularly in its protein composition. This study delves into the detection and quantification of cardiac damage markers such as CK-MB and myoglobin in vitreous humor samples from 45 autopsy cases. For the first time, it demonstrates a statistically significant correlation between these markers and the perimortem agony interval (PAI), defined as the survival time before death. This discovery paves the way for innovative forensic applications, including the estimation of the PAI, a critical parameter for judicial and compensatory assessments. The findings underscore the potential of the vitreous humor as a diagnostic medium, opening new avenues for understanding the systemic dynamics of cardiac markers and the role of the blood–retinal barrier in post-mortem scenarios.

## 1. Introduction

The vitreous humor, the gel-like substance occupying the space between the lens and the retina in the eye, is composed primarily of water (approximately 98–99%), with the remainder consisting of a network of structural proteins, polysaccharides, and soluble metabolites [1,2,3]. The primary structural component is hyaluronic acid, a glycosaminoglycan that provides the vitreous with its viscoelastic properties and contributes to its transparency. Collagen fibrils, predominantly type II collagen, form a scaffold that supports the gel matrix. In addition to these macromolecules, the vitreous humor contains various low-molecular-weight solutes, including electrolytes (sodium, potassium, and calcium), glucose, and lactate, which are essential for maintaining cellular metabolism and osmotic balance within the eye. The biochemical composition of the vitreous humor reflects its role as a reservoir for metabolites and a medium for molecular exchange between the retina and surrounding tissues. Due to its isolation from direct vascular supply, changes in its biochemical profile often mirror systemic physiological or pathological states. Forensic investigations have capitalized on this property, utilizing the vitreous humor to detect markers of systemic conditions such as electrolyte imbalances, hyperglycemia, and uremia. Its stability post-mortem, relative to other body fluids, further enhances its utility in forensic applications, particularly in evaluating time-dependent biochemical changes [4,5,6].

Determining the cause of death remains one of the most complex challenges in forensic medicine, especially in cases of sudden or agonal death. Traditional methods of post-mortem investigation, such as histopathology and toxicology, have proven invaluable, yet they often fail to provide sufficient insight into the systemic physiological changes occurring during the perimortem interval. Recent advances in forensic biochemistry have highlighted the potential of the vitreous humor as a unique biofluid for post-mortem analysis [7,8]. Protected from environmental contamination and systemic redistribution, the vitreous humor offers a stable medium for the detection of biochemical markers. Cardiac markers, including CK-MB, myoglobin (MYO), troponin I (TnI), brain natriuretic peptide (BNP), and D-dimer, are well-established indicators of myocardial stress and damage in clinical settings [9,10,11]. Their role in forensic investigations, particularly when sampled from the vitreous humor, remains underexplored. Unlike blood or urine, the vitreous humor is less susceptible to post-mortem changes and retains its biochemical profile for extended periods, making it a reliable medium for post-mortem biochemical analysis. Understanding the behavior of these markers in the vitreous humor could provide valuable insights into the physiological state of the individual during the perimortem period, particularly in cases involving prolonged agony or systemic distress.

The perimortem agony interval (PAI), defined as the survival period during which systemic stress precedes death, represents a critical aspect of forensic investigations. The agonal phase, which represents the final moment before death, is marked by a series of profound physiological and biochemical changes as the body gradually shuts down. During this critical period, the body experiences the failure of vital systems, such as the respiratory, circulatory, and nervous systems. As a result, a cascade of biochemical events takes place, many of which can provide valuable insights for forensic investigations. One of the most significant changes that occurs is the disruption of cellular homeostasis. With the cessation of blood circulation, oxygen delivery to tissues becomes impaired, and cells are forced to rely on anaerobic metabolism. This shift leads to the accumulation of lactic acid in tissues and fluids, reflecting the body’s struggle to produce energy without the presence of sufficient oxygen. Elevated lactate levels can indicate that the body was under significant stress during the agonal phase, which may be a key factor in determining the cause of death, especially in cases where there is evidence of ischemia or oxygen deprivation.

This study seeks to bridge the gap in forensic biochemistry by examining the correlation between cardiac markers in the vitreous humor and agonal death. A cohort of 45 autopsy cases was analyzed, with samples categorized based on the presence or absence of measurable cardiac markers. The findings aim to elucidate the dynamics of biomarker migration into the vitreous humor and their potential forensic applications. By combining biochemical analysis with statistical modeling, this research provides a novel approach to understanding systemic changes in agonal and sudden deaths. The implications of these findings extend beyond forensic medicine. They contribute to a deeper understanding of the physiological processes associated with death, offering insights that could inform both clinical and judicial contexts. The ability to reliably estimate the PAI and identify conditions associated with systemic stress opens new avenues for forensic diagnostics, enabling more precise determinations of the circumstances surrounding death.

## 2. Results

### 2.1. Statistical Analysis

For age, both groups showed a normal distribution, allowing the calculation of means and standard deviations. Specifically, the mean age for the “Agonizing Death” group was 39.8 years (SD = 25.7), while for the “Sudden Death” group, it was 47.6 years (SD = 21.3). For the post-mortem interval (PMI), the “Agonizing Death” group did not meet the assumption of normality, while the “Sudden Death” group did. Therefore, the median and interquartile range (IQR) were reported for the PMI: the median PMI was 72 h (IQR = 37) for the “Agonizing Death” group and 97 h (IQR = 42.8) for the “Sudden Death” group. Summary statistics for categorical variables were obtained using frequency tables with cross-tabulations. The resulting table containing the descriptive statistics is presented in Table 1.

The results show a significant association between the groups (“Agonizing Death” and “Sudden Death”) and the biomarkers analyzed, with varying magnitudes depending on the marker.

For the CK-MB biomarker, as we can see from Table 2, all subjects in the “Sudden Death” group tested negative, while 100% of the “Agonizing Death” group tested positive. The association analysis using the Phi statistic revealed a very large effect (ϕ = 0.99, 95% CI [0.74, 1.00]).

For the MYO biomarker, most subjects in the “Agonizing Death” group (15 out of 17) tested positive (Table 3), while all subjects in the “Sudden Death” group tested negative. Here, too, the observed effect was classified as very large (ϕ = 0.89, 95% CI [0.64, 1.00]).

Regarding the TnI biomarker, the results were less defined: fifteen subjects in the “Agonizing Death” group tested negative and two tested positive, while all subjects in the “Sudden Death” group tested negative (Table 4). Although the Phi statistic indicated a medium effect (ϕ = 0.23, 95% CI [0.00, 1.00]), it is important to note that the small sample size and the wide confidence interval suggest caution in interpreting these results.

Similarly, for the D-dimer biomarker, three subjects in the “Agonizing Death” group tested positive compared to none in the “Sudden Death” group (Table 5). The observed effect was classified as large (ϕ = 0.31, 95% CI [0.00, 1.00]); however, the wide confidence interval and the low number of positive cases may reflect uncertainty in the association.

Finally, for the BNP biomarker, all participants in both groups tested negative (Table 6). This result indicates no significant variation.

### 2.2. Evaluation of Results

Overall, the results suggest a strong association between certain biomarkers (CK-MB and MYO) and the analyzed groups. For other markers (TnI and D-dimer), the observed association could be influenced by the limited sample size and the wide confidence intervals, warranting further investigation for a more accurate evaluation.

The analysis of the 45 cases revealed distinct differences in the biochemical profiles of the vitreous humor between cases with measurable cardiac markers and those without. In cases where no markers were detected, the CK-MB, myoglobin, troponin I, BNP, and D-dimer levels consistently remained below the thresholds of detection. These cases were predominantly associated with rapid deaths due to acute traumatic injuries or sudden cardiovascular events. The rapidity of these deaths likely precluded the migration of systemic markers into the vitreous humor, consistent with the hypothesis that time and systemic permeability are critical factors influencing biomarker accumulation.

In contrast, cases with detectable levels of cardiac markers exhibited a wide range of concentrations, particularly for CK-MB and myoglobin. These findings were strongly associated with prolonged perimortem agony intervals, where systemic stress and organ failure likely disrupted the blood–retinal barrier, allowing cardiac markers to infiltrate the vitreous humor. For example, myoglobin levels ranged from baseline values to over 200 ng/mL, while D-dimer levels frequently exceeded 100 ng/mL in cases of systemic thrombotic activity. These elevated levels were indicative of significant systemic stress and were closely correlated with extended survival times before death.

The comparison between cases with and without detectable markers highlighted the role of the perimortem agony interval in biomarker migration. Prolonged agony was not only associated with higher marker levels but also with greater variability, reflecting the diverse physiological responses of death, particularly in distinguishing between rapid deaths and those characterized by extended systemic distress, progressive organ failure, and prolonged hypoxic and inflammatory states, which contribute to differential biomarker accumulation in the vitreous humor.

## 3. Discussion

The findings of this study provide compelling evidence for the utility of vitreous humor analysis in forensic investigations, particularly in understanding the physiological changes associated with the perimortem agony interval. The presence of cardiac markers such as CK-MB and myoglobin in the vitreous humor underscores the systemic stress and myocardial damage that occur during prolonged agony. This study confirms that the migration of these markers into the vitreous humor is a time-dependent process, influenced by systemic factors such as hypoxia, acidosis, and inflammation [6,7,8,9].

Hypoxia, a hallmark of the perimortem interval, plays a pivotal role in disrupting the blood–retinal barrier (BRB). Under normal physiological conditions, the BRB maintains the homeostasis of the retinal environment, preventing the influx of systemic molecules. However, hypoxia triggers a cascade of events that compromise the integrity of this barrier. Elevated levels of vascular endothelial growth factor (VEGF), a potent mediator of vascular permeability, have been implicated in hypoxia-induced BRB disruption. Studies demonstrate that VEGF promotes the disassembly of tight junction proteins, including occludins and claudins, which are essential for maintaining BRB integrity. The breakdown of these tight junctions facilitates the migration of macromolecules, including cardiac markers, into the vitreous humor [10,11,12].

Inflammation further exacerbates BRB permeability during systemic stress. The release of pro-inflammatory cytokines, such as tumor necrosis factor-alpha (TNF-α) and interleukin-6 (IL-6), disrupts endothelial cell tight junctions and alters the cytoskeletal structure. The combination of hypoxia-induced VEGF release and cytokine-driven inflammation creates a permissive environment for the diffusion of biomarkers into the vitreous humor. This effect is further amplified in systemic conditions involving sepsis or prolonged hypoxia [10,11,12,13,14,15,16].

Acidosis, commonly observed in the perimortem period, also contributes to BRB dysfunction. Studies have shown that acidic environments destabilize endothelial cell membranes, impairing the BRB. This destabilization is exacerbated by systemic lactic acidosis, a byproduct of prolonged hypoxia and ischemia, which is often observed in cases of prolonged agony. The role of acidosis is particularly significant when coupled with inflammatory mediators, as the synergistic effects can amplify BRB permeability. These findings are supported by clinical observations in patients with acute respiratory distress syndrome (ARDS), where systemic hypoxia and acidosis correlate with increased vascular permeability, including in retinal vasculature.

**Figure 1:**
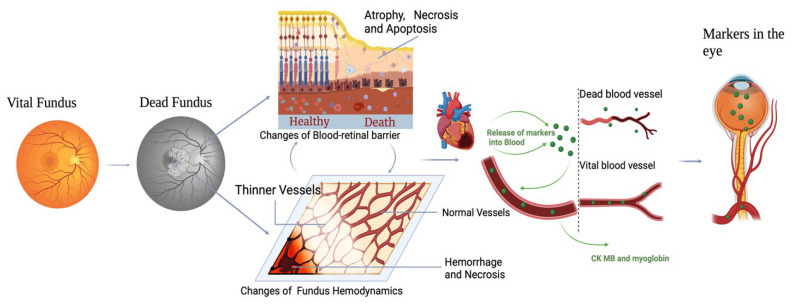
Illustration of the alteration mechanism of the blood–retinal barrier (BRB) during the perimortem period. The figure highlights the increased BRB permeability under conditions of hypoxia, acidosis, and inflammation, facilitating the passage of cardiac markers into the vitreous humor.

A novel hypothesis emerging from this study is the potential role of an inflammatory storm in altering the integrity of the blood–retinal barrier (BRB). During prolonged systemic stress or agony, the body often enters a hyperinflammatory state characterized by the release of a cascade of pro-inflammatory cytokines, including tumor necrosis factor-alpha (TNF-α), interleukin-6 (IL-6), and interleukin-1 beta (IL-1β). This cytokine storm can have profound effects on endothelial integrity, particularly in highly vascularized and sensitive areas such as the retina. In the context of vitreous humor analysis, this inflammatory storm likely acts as a key driver for the migration of cardiac markers such as CK-MB and myoglobin. The synergistic effects of hypoxia-induced VEGF release, systemic acidosis, and inflammation create an environment where the BRB becomes permeable. This permeability not only allows the diffusion of systemic biomarkers but also underscores the critical interplay of local and systemic factors in the biochemical changes observed during the perimortem interval [10,11,12,13,14,15,16] (Figure 1).

**Figure 2:**
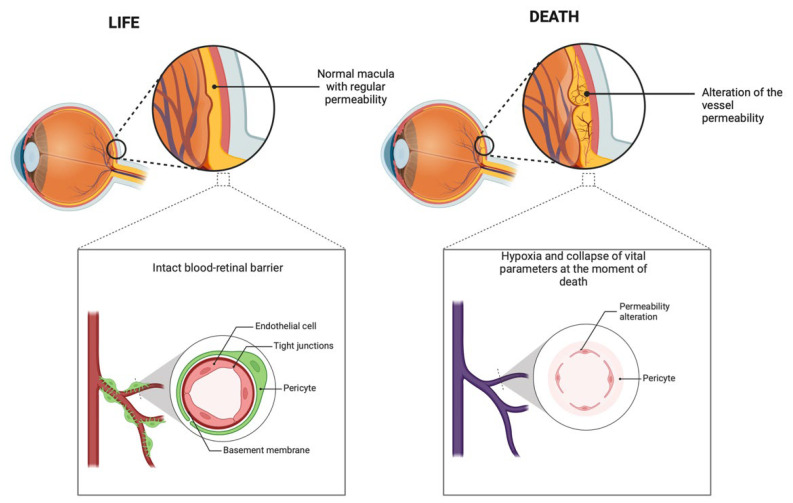
Schematic representation of BRB modifications in response to systemic factors such as hypoxic stress and inflammation. The increased permeability promotes the migration of plasma biomarkers into the vitreous humor, with implications for forensic analysis.

While certain cardiac markers, such as CK-MB and myoglobin, show significant variability between the positive and negative groups, others, including troponin I and BNP, exhibit relatively stable levels across cases. This consistency may be attributed to several factors. First, the size and molecular characteristics of these markers influence their ability to migrate through the compromised BRB. For example, larger molecules or those with specific binding affinities to plasma proteins may not diffuse as readily into the vitreous humor, even under conditions of increased permeability. Second, the systemic concentration of certain markers at the time of death might not reach the threshold necessary for detectable migration. Troponin I, for instance, is highly specific to myocardial injury and is released in smaller quantities compared to myoglobin. As such, in cases where myocardial injury is less severe or absent, the systemic levels of troponin I may remain below the detectable range in the vitreous humor (Figure 2).

The variability observed in biomarker levels among cases with prolonged agony underscores the complexity of interpreting vitreous humor biochemistry. Individual differences in physiological responses, including the extent of systemic inflammation and the duration of hypoxia, likely contribute to this variability. Elevated BNP levels in some cases were indicative of chronic cardiac stress, suggesting that pre-existing conditions such as heart failure or hypertension may influence biomarker dynamics. While the detection of cardiac markers in the vitreous humor provides valuable forensic insights, it is crucial to consider potential confounding factors. The post-mortem redistribution of biomarkers, although less prominent in the vitreous humor compared to blood, may still influence marker levels. This phenomenon underscores the importance of correlating biomarker data with clinical history and environmental factors to ensure accurate interpretation.

From a forensic perspective, the ability to correlate cardiac marker levels with the perimortem agony interval offers a promising tool for reconstructing the sequence of events leading to death. The stability of the vitreous humor as a biofluid, combined with its relative isolation from post-mortem redistribution processes, makes it an ideal medium for such analyses. Furthermore, the detection of elevated D-dimer levels in cases with prolonged agony highlights the potential role of systemic coagulation and thrombotic activity as secondary markers of systemic stress. Despite its potential, this study has several limitations. The sample size, though sufficient for preliminary analysis, limits the generalizability of the findings. Future studies with larger cohorts are needed to validate these results and explore additional biomarkers that may offer further insights into systemic changes during the perimortem interval. Additionally, the observational design of this study precludes experimental control over confounding variables. Controlled experimental studies, particularly those simulating perimortem conditions, could provide a deeper understanding of the mechanisms driving biomarker migration into the vitreous humor. Advances in proteomics and metabolomics may also enhance the sensitivity and specificity of vitreous humor analysis, allowing for the detection of novel biomarkers associated with systemic stress and organ failure.

Finally, while this study focused primarily on cardiac markers, the integration of other systemic markers, such as lactate, glucose, and cytokines, could provide a more comprehensive view of the biochemical changes occurring during the perimortem period. By combining these markers with advanced imaging techniques, such as mass spectrometry imaging, future research could further elucidate the spatial and temporal dynamics of biomarker migration within the vitreous humor [16,17,18].

## 4. Materials and Methods

This study was conducted at the Institute of Legal Medicine, University Magna Graecia of Catanzaro, over a year-long period from March 2023 to March 2024. Ethical approval was secured from the Ethics Committee of Regione Calabria under protocol code [no. 69 of 26 February 2024]. A total of 45 autopsy cases were selected based on rigorous inclusion criteria. These criteria required that each case had sufficient amounts of the vitreous humor available for analysis, a documented and reliable time of death, and an absence of contamination in the collected samples. Exclusion criteria included significant decomposition or insufficient vitreous humor volume.

The vitreous humor samples were extracted during routine autopsies following strict sterile protocols. Each sample was collected using an 18-gauge needle attached to a 5 mL syringe, which was inserted into the posterior chamber of the eye. Approximately 2 mL of the vitreous humor was aspirated, ensuring the sample was free from blood contamination. All samples were transferred immediately to sterile containers and stored at 4 °C. Biochemical processing occurred within 24 h to preserve sample integrity.

**Figure 3:**
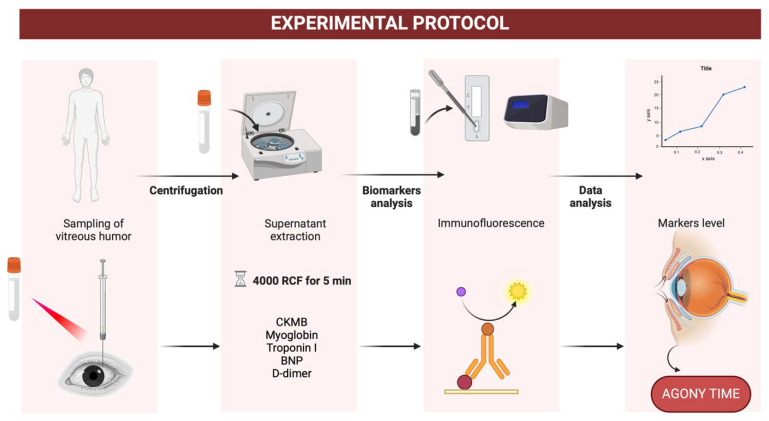
Diagram of the experimental protocol used in this study. The key steps are illustrated, including vitreous humor sampling, sample preparation, and biochemical analysis of cardiac markers using immunoassays.

The biochemical analysis was conducted using the Quidel Triage Profiler SOB Panel. This immunoassay platform facilitated the quantification of five key cardiac markers: CK-MB, myoglobin (MYO), troponin I (TnI), brain natriuretic peptide (BNP), and D-dimer. Each marker was selected for its clinical relevance in reflecting cardiac stress, muscle injury, or systemic coagulation. Samples were first centrifuged at 4000 g for five minutes to separate cellular debris. The supernatant was then diluted in sterile saline at a 1:2 ratio to enhance compatibility with the assay. Measurements were performed in duplicate to ensure accuracy, with results reported in ng/mL for CK-MB, MYO, TnI, and D-dimer and pg/mL for BNP (Figure 3).

We enrolled 45 subjects: 17 classified as Agonizing Death and 28 as Sudden Death. We collected various variables, like age, sex, Post Mortem Index (PMI), Causa Mortis, CK-MB, MYO, TnI, BNP, D-dimer, and Perimortem Agony Interval (PAI). We calculated the appropriate descriptive statistics: mean and standard deviation for approximately normal distribution or median and IQR for non-normal distribution. To assess normality, we plotted the distribution of quantitative variables using histograms and the Shapiro–Wilk normality test, considering the variable normally distributed when the Shapiro–Wilk *p*-value > 0.05. To verify if there was an association between being positive to a specific biomarker and dying from an agonizing or sudden death, we first reported each frequency into a 2 × 2 contingency table, and then we calculated the appropriate effect size index. In this case, we did not always choose to conduct a Chi-Squared Test because some cells were empty. We preferred to use phi (ϕ) effect size to quantify the correlation between two dichotomous variables and to interpret it following Funder and Ozer (2019) rules [19]. Inferential tests were conducted only if all the cells contained at least one frequency. All analyses were conducted with R version 4.3.0. *p*-values below an alpha level of 0.05 were considered statistically significant.

Comprehensive autopsies were performed on all cases. These involved external examination and systematic internal evaluations of the cranial, thoracic, and abdominal cavities. Organs were excised, weighed, and sampled for histopathological analysis. Hematoxylin–eosin staining was utilized to identify pathological changes such. Toxicological screenings were conducted on peripheral blood and urine samples to detect the presence of exogenous substances and focused on identifying drugs, alcohol, or other psychoactive compounds.

The data were analyzed statistically to explore relationships between cardiac marker levels, the post-mortem interval (PMI), and the PAI. Descriptive statistics summarized the data distribution, including mean, median, standard deviation, and range. Pearson’s correlation coefficient was calculated to evaluate the strength of associations between biomarkers and the PMI or PAI. Comparative analysis using independent *t*-tests assessed differences in marker levels between cases with detectable and undetectable markers. Multivariate regression models further explored the predictive value of cardiac markers for estimating the PAI while accounting for confounders such as age, sex, and PMI. Statistical significance was set at *p* < 0.05, and the results are presented with corresponding confidence intervals (Table 7 and Table 8).

## 5. Conclusions

This study highlights the significance of the vitreous humor as a medium for post-mortem biochemical investigations, demonstrating its potential to reflect systemic changes associated with the perimortem agony interval. The detection and quantification of cardiac markers such as CK-MB, myoglobin, and D-dimer provide valuable insights into the physiological processes underlying prolonged systemic stress and myocardial damage. These findings not only advance the understanding of biomarker dynamics in the vitreous humor but also establish a foundation for forensic applications aimed at estimating the perimortem agony interval. The correlation between cardiac markers and systemic stress underscores the importance of integrating biochemical analysis into routine forensic investigations. By combining vitreous humor analysis with traditional autopsy findings, forensic practitioners can achieve a more comprehensive understanding of the circumstances surrounding death. However, further research is essential to refine the diagnostic accuracy of these methods, particularly in cases with confounding variables, such as pre-existing medical conditions or environmental factors influencing post-mortem intervals. In conclusion, this study underscores the potential of the vitreous humor as a diagnostic biofluid in forensic medicine. By providing a window into the systemic changes that precede death, it offers a valuable tool for reconstructing the sequence of events leading to mortality. Continued research in this area promises to enhance the accuracy and reliability of forensic diagnostics, ultimately contributing to more precise and informed medico-legal determinations.

## Figures and Tables

**Table 1:** Descriptive statistics.

	Agonizing	Sudden
N	17	28
Age	39.8 (25.7) ^†^	47.6 (21.3) ^†^
Sex	6 F	8 F
	11 M	20 M
PMI	72 (37) *	97 (42.8) *

^†^ values are reported as mean (standard deviation); * median (IQR).

**Table 2:** Contingency table for CK-MB.

	Negative	Positive
Agonizing	0	17
Sudden	28	0

**Table 3:** Contingency table for MYO.

	Negative	Positive
Agonizing	2	15
Sudden	28	0

**Table 4:** Contingency table for TnI.

	Negative	Positive
Agonizing	15	2
Sudden	28	0

**Table 5:** Contingency table for D-dimer.

	Negative	Positive
Agonizing	14	3
Sudden	28	0

**Table 6:** Contingency table for BNP.

	Negative	Positive
Agonizing	17	0
Sudden	28	0

**Table 7:** Cases with sudden death.

Sex	Age	PMI	Cause of Death	CK-MB	MYO	TnI	BNP	D-Dimer
M	28	183 h	Traumatic shock from train accident	<1.0	<5.0	<0.05	<5.0	<100
M	48	50 h	Traumatic shock from falling	<1.0	<5.0	<0.05	<5.0	<100
M	16 months	70 h	Hypovolemic shock	<1.0	<5.0	<0.05	<5.0	<100
M	52	99 h	Arrhythmia from myocardial infarction	<1.0	<5.0	<0.05	<5.0	<100
M	54	108 h	Arrhythmia from myocardial infarction	<1.0	<5.0	<0.05	<5.0	<100
M	71	95 h	Arrhythmia from myocardial infarction	<1.0	<5.0	<0.05	<5.0	<100
M	50	75 h	Arrhythmia from myocardial infarction	<1.0	<5.0	<0.05	<5.0	<100
F	72	45 h	Hanging	<1.0	<5.0	<0.05	<5.0	<100
F	86	132 h	Pulmonary thromboembolism	<1.0	<5.0	<0.05	<5.0	<100
F	79	167 h	Arrhythmia from myocardial infarction	<1.0	<5.0	<0.05	<5.0	<100
M	68	194 h	Arrhythmia from myocardial infarction	<1.0	<5.0	<0.05	<5.0	<100
M	69	135 h	Arrhythmia from myocardial infarction	<1.0	<5.0	<0.05	<5.0	<100
M	43	96 h	Spinal shock due to C2 fracture	<1.0	<5.0	<0.05	<5.0	<100
M	33	120 h	Arrhythmia from myocardial infarction	<1.0	<5.0	<0.05	<5.0	<100
M	61	96 h	Traumatic shock from falling	<1.0	<5.0	<0.05	<5.0	<100
M	13	66 h	Massive head trauma	<1.0	<5.0	<0.05	<5.0	<100
M	46	168 h	Arrhythmia from myocardial infarction	<1.0	<5.0	<0.05	<5.0	<100
M	31	97 h	Arrhythmia from myocardial infarction	<1.0	<5.0	<0.05	<5.0	<100
M	37	192 h	Pulmonary thromboembolism	<1.0	<5.0	<0.05	<5.0	<100
F	60	97 h	Arrhythmia	<1.0	<5.0	<0.05	<5.0	<100
F	34	96 h	Traumatic shock from car accident	<1.0	<5.0	<0.05	<5.0	<100
M	27	96 h	Traumatic shock from car accident	<1.0	<5.0	<0.05	<5.0	<100
F	24	96 h	Traumatic shock from car accident	<1.0	<5.0	<0.05	<5.0	<100
M	38	16 h	Arrhythmia	<1.0	<5.0	<0.05	<5.0	<100
M	68	120 h	Traumatic shock from car accident	<1.0	<5.0	<0.05	<5.0	<100
F	75	264 h	Heart rupture with hemopericardium	<1.0	<5.0	<0.05	<5.0	<100
F	43	56 h	Arrhythmia from myocardial infarction	<1.0	<5.0	<0.05	<5.0	<100
M	23	129 h	Traumatic shock from car accident	<1.0	<5.0	<0.05	<5.0	<100

**Table 8:** Cases with agonal death.

Sex	Age	PMI	Cause of Death	CK-MB (ng/mL)	MYO (ng/mL)	TnI (ng/mL)	BNP (pg/mL)	D-Dimer (ng/mL)
F	22	50 h	Prolonged ventricular fibrillation	>80.0	124	<0.05	<5.0	100
F	21	96 h	Polytrauma with liver and lung lacerations	>80.0	17.3	<0.05	<5.0	<100
M	2	72 h	Asphyxia due to pneumonia	>80.0	51.0	<0.05	<5.0	<100
M	51	46 h	Stabbing homicide with lung injuries	60.9	17.8	<0.05	<5.0	<100
F	0	153 h	Intrauterine fetal death	>80.0	83.2	<0.05	<5.0	<100
M	33	96 h	Septic shock	>80.0	170	0.12	<5.0	<100
M	46	336 h	Hemorrhagic shock with lung injuries from gunshot	>80.0	212	<0.05	<5.0	172
M	54	72 h	Polytrauma from fall with subsequent hospital death	>80.0	233	<0.05	<5.0	<100
M	62	96 h	Myocardial infarction with macroscopic signs	11.5	14.6	<0.05	<5.0	<100
M	26	72 h	Asphyxia from drowning	11.7	169	0.21	<5.0	1910
M	74	50 h	Asphyxia from anaphylactic shock	55.3	40.9	<0.05	<5.0	<100
M	39	68 h	Gunshot homicide with lung injuries	26.6	<5.0	<0.05	<5.0	<100
F	35	41 h	Asphyxia from hanging	18.2	<5.0	<0.05	<5.0	<100
F	23	129 h	Asphyxia from inhalation of pulmonary blood	>80.0	22.0	<0.05	<5.0	<100
M	94	86 h	Myocardial infarction with macroscopic signs	52.4	20.6	<0.05	<5.0	<100
M	72	59 h	Polytrauma from fall from medium height	>80.0	247	<0.05	<5.0	<100
F	22	60 h	Asphyxia from hanging	78.2	16.7	<0.05	<5.0	<100

## Data Availability

Data are contained within the article.
